# The Role of Technology in Adherence to Physical Activity Programs in Patients with Chronic Diseases Experiencing Fatigue: a Systematic Review

**DOI:** 10.1186/s40798-019-0214-z

**Published:** 2019-09-12

**Authors:** Andrea Albergoni, Florentina J. Hettinga, Antonio La Torre, Matteo Bonato, Francesco Sartor

**Affiliations:** 1 Department of Biomedical Sciences for Health, Università degli Studi di Milano, Milan, Italy; 2 Department of Patient Care & Measurements, Philips Research, Eindhoven, The Netherlands; 3 School of Sport Rehabilitation and Exercise Sciences, University of Essex, Colchester, UK; 4 Department of Sport, Exercise and Rehabilitation, Northumbria University, Newcastle, UK; 5 IRCCS Istituto Ortopedico Galeazzi, Milan, Italy; 6 College of Health & Behavioural Science, Bangor University, Bangor, UK; 7 Philips Electronics Nederland B.V, HTC 34 1.011, P.O. Box WB61, 5656 AE Eindhoven, The Netherlands

**Keywords:** Guidelines, Patients, Technology, Wearables, Pedometers, Activity monitors, Cancer, COPD, Self-efficacy steps

## Abstract

**Background:**

The beneficial role of physical activity (PA) to manage the health condition of patients with chronic diseases is well known. However, adherence to PA guidelines in this group is still low. Monitoring and user-interface technology could represent a significant tool to increase exercise adherence to those particular groups who experience difficulties in adhering to regular and substantial physical activity, and could be supportive in increasing the success of PA programs and interventions. This systematic review aimed at evaluating the effect of physical activity monitoring technology in improving adherence to a PA program in patients with chronic diseases experiencing fatigue.

**Methods:**

This systematic review was conducted according to PRISMA guidelines. The literature search was performed in Embase, Medline, Biosis, Scopus, and SPORTDiscus. We filtered the literature according to the question: “Does monitoring technology affect adherence to physical activity and exercise programs in patients with chronic diseases perceiving fatigue?”.

**Results:**

The search resulted in 1790 hits; finally, eight studies were included, with a total number of 205 patients. Study quality was moderate except for one study of high quality. Only three disease types emerged, COPD, HF, and cancer. PA programs were rather short (from 8 to 13 weeks) except for one 3-year-long study. Five studies employed pedometers and two an activity monitor. Three studies based their adherence on steps, the remaining studies focused on active minutes. Adherence was explicitly reported in two studies, and otherwise derived. Four studies showed high adherence levels (85% week-10, 89% week-8, 81% week-13, 105% week-13, 83% average week-1–12) and three low levels (56% week-12, 41% year-2, 14 year-3).

**Conclusion:**

The small number of studies identified did not allow to establish whether the use of monitoring technology could improve adherence to PA programs in patients with chronic diseases experiencing fatigue, but the current evidence seems to suggest that this is a field warranting further study, particularly into how monitoring technology can help to engage patients to adhere to PA programs.

## Key Points

- Although monitoring technology is a clear emerging trend in promoting physical activity in patients with chronic diseases and has potential, hitherto there is not enough evidence to clarify if the use of technology supports patients with chronic diseases to increase exercise adherence.
- Technology is mainly used to monitor physical activity, but not yet to improve exercise adherence. In the few studies where this was the case, adherence levels were high. The role of fatigue needs to be further researched and the definition of adherence needs to be standardized.

## Background

There is a large body of evidence showing the benefit of physical activity (PA) and physical exercise for patients with chronic diseases [1]. The concept “exercise is medicine” as defined by the American College of Sports Medicine (ACSM), has been widely accepted for the prevention, and in some specific cases, for the treatment of chronic diseases such as cancer, type 2 diabetes mellitus (T2DM), cardiovascular diseases [2], and of people with disabilities [3]. In fact, PA and physical exercise have been considered a real “polypill” in primary as well as secondary prevention [4, 5]. Since physical exercise can be considered a subcategory of PA [1], we will here include physical exercise in PA. Leisure-time activity, low, moderate, and vigorous activities have been linked to a reduction in the risk of T2DM [6], and in inflammatory markers in breast cancer survivors [7]. In cancer survivors, PA improves quality of life, cardiorespiratory fitness and strength, and it alleviates fatigue [8, 9]. In chronic obstructive pulmonary disease (COPD) patients, PA is associated with better respiratory parameters (e.g., FEV_1_, dyspnea) [10]. PA has a strong effect in reducing atherosclerotic factors, typical of cardiovascular diseases [11]. Moderate-to-vigorous PA bouts were associated with lower severity of pain and fatigue in women with fibromyalgia [12], and graded exercise therapy has shown benefits for myalgic encephalomyelitis patients [13]. According to a recent meta-analysis, exercise rehabilitation improved exercise capacity as well as health-related quality of life of heart failure patients and it should be offered to all heart failure patients [14]. In patients with chronic psychological disorders, PA helps to increase self-esteem and to reduce depression [15].

Despite the fact that this evidence shows the importance of PA in preventing and treating patients with chronic diseases, the adherence to guidelines is still rather low. Patients with a serious mental illness were less active than the general population [15], only the 9% of them reached the PA guidelines [16]. Hartman et al. (2010) found that in Sweden, 84% of COPD, 74% of rheumatoid arthritis, 72% of T2DM and, 60% of healthy individuals did not adhere to PA guidelines. In a UK cohort of seniors, only 15% of men and 10% of women met guidelines [17] and dramatically less than 5% of adults in the USA [18].

The term adherence when referring to PA is not always well defined and uniform. Adherence to a program can be intended as the number of sessions conducted over the total number of sessions prescribed, either accounting for, or regardless whether the sessions were fulfilled or not [19]. For instance, a patient exercising three times a week, 30 min per session, as prescribed, would have an adherence of 100% as well as a patient training three times a week but only for 15 min per session, if only the number of sessions performed were to be accounted for.

The major factors that hinder exercise adherence in patients with chronic diseases are low self-efficacy, depression, anxiety, helplessness, poor social support or activity, greater perceived number of barriers to exercise, and increased pain levels during exercise [20]. In addition to these, fatigue in patients with chronic diseases is a common symptom that decreases adherence to a PA program [21]. In patients with chronic diseases, fatigue, defined as “a subjective feeling of tiredness, weakness or lack of energy” [22], is reported to be a major obstacle to PA execution [23]. Indeed, fatigue is an important factor related with low levels of PA in COPD patients [24]. Fatigue is a typical symptom in cancer patients [25] and it persists even after chemotherapy [9]. Fatigue is also a common symptom in T2DM patients [26], and PA has been shown to help to manage it [27]. Muscular pain and fatigue make PA intolerable in many chronic heart failure (HF) patients [28]. Recently, it has been found that activity pacing, a strategy to divide one’s daily activities into more manageable portions, might have sustained beneficial effects on management and reduction of fatigue in persons with disabilities or chronic diseases associated with fatigue complaints [29].

In the past years, there has been a large number of strategies aiming at lowering the barriers to engage in PA programs. It is now common for patients to discuss their PA habits with their health professionals [30]. Behavioral interventions such as motivational interviewing and goal-setting are commonly used [31]. Furthermore, large-scale strategies, such as national walking programs, have been also deployed to increase PA in the entire population [32]. Recently, programs also started targeting persons with disabilities and chronic diseases for example in the ReSpAct study in the Netherlands [33]. Finally, health professional supervision by itself can increase adherence, and monitoring is its key element [34].

Electronic devices can be adopted to monitor adherence as long as they are used in a systematic manner [34]. However, there is a large gap between self-reported PA and measured PA [35] as self-reported PA is known to be not always reliable. For this reason, PA programs better be implemented with the use of human interface and monitoring technology such as web sites [30, 32, 36], mobile devices [37], apps [38], and wearable devices [39]. Additionally, the presence of an objective goal (daily steps or exercise minutes) can be a key-factor to successfully increase PA, without it the improvements may be limited or even absent [40]. Wearable technology was identified as the leading fitness trend in 2019 [41]. Indeed, ACSM-certified professionals identified it as a tool to positively change PA behavior [41]. Activity and exercise metrics such as step counts, distance covered, active or walking time can be tracked by pedometers, in addition, more recently, energy expenditure, activity types, and intensity can be monitored by accelerometer and heart rate sensors implemented on wearables or, in some cases, on smartphones. Nowadays, also global positioning systems are often used for physical and exercise activity tracking [42]. The added value of technology is not confined to monitoring patients’ PA objectively, but it extends to patients’ stimulation and engagement to increase adherence [43]. For instance, human interface technology can reduce the gap between therapists and patients, who may feel more responsible to adhere when directly supervised [44]. Furthermore, technology tools offer the possibility for a more enjoyable and motivational approach to PA programs (e.g., exergaming) [44, 45]. Taken together, this evidence suggests the high potential of the monitoring and user-interface technology in promoting an active lifestyle in persons with disabilities and chronic diseases.

The aim of this systematic review was to evaluate whether in patients with chronic diseases experiencing fatigue the use of monitoring technology would improve adherence to a PA program in the mHealth and eHealth space such as a home and/or rehabilitation settings.

## Methods

This systematic review was conducted following PRISMA’s guidelines [46], and it was registered in the Prospero Database (CRD42018109081). The research question for this systematic review was: “Does monitoring technology affect adherence to physical activity and exercise programs in patients with chronic diseases perceiving fatigue?”. Studies published in English from the year 2006 were included in the search. We did not exclude conference abstracts and requested full-texts when they were not available. We collected studies from Embase, Medline, and Biosis on the 19^th^ of March 2018, using this keywords (((((((physical n1 activ* OR function*) n10 program*) OR (activity n2 pacing) OR rehabilitation) AND (adhere OR motiv* OR complain*))AND (pedometer* OR accelerometer* OR monitor* OR wearable* OR watch* OR phone* OR band* OR heart rate* OR monitor* OR telemonitor* OR mhealth* OR ehealth* OR telehealth*)) AND ((chronic* n5 disease* OR ill*) OR copd OR fatigue OR diabetes OR heart failure OR cancer OR malignant neoplasm OR osteoarthritis OR rheumatoid arthritis OR fibromyalgia OR cancer fatigue OR chronic fatigue syndrome)))). An additional search in the same databases was executed on the 23^rd^ of March 2018 adding “cancer” to the previous search. The search was executed using Scientific & Technical Information Network library; additionally, three more searches were conducted with the same key words in the same database as mentioned above with the inclusion of Scopus, and SPORTDiscus the 30^th^ of April 2019.

In order to be included, the studies had to satisfy the following criteria: Was this study conducted in a clinical population experiencing fatigue? Was monitoring technology used? Did this study concern activity/exercise programs? Was adherence calculated or derivable from this study? Was monitoring technology used to improve adherence? The use of monitoring technology in improving adherence referred to continuous and long at least one month period. Studies in which monitoring technology was used, but not to provide any feedback, were excluded. For instance, studies in which participants were blinded to pedometers or accelerometers output were not included. Full text screening was conducted by two independent researchers (A.A. and F.S.).

Adherence was generally defined as PA performed over the total PA target prescribed times 100. If adherence was not directly available, we calculated it.

Quality was assessed according to the Downs and Black checklist [47] cited in the Cochrane’s Handbook for Systematic Reviews of Interventions (see Table 1) [48]. We assigned a score of 1 in case of a positive answer, 0 when negative or unknown. Studies with a total score from 11 to 15 were considered “high quality”, 6–10 “medium quality”, 0–5 “low quality.” Two studies scored high quality [49, 50], the other studies were classified as medium quality (Table 2).

**Table 1:** Quality assessment questions

1	Is the hypothesis/aim/objective of the study clearly described?
2	Are the characteristics of the patients included in the study clearly described?
3	Are the main findings of the study clearly described?
4	Does the study provide estimates of random variability in the data for the main outcomes (e.g., interquartile range for non-normally distributed data; standard error, standard deviation, or confidence intervals for normally distributed data)?
5	Have the actual probability values been reported (e.g., .035 rather than < .05) for the main outcomes except where the probability value is less than .001?
6	Were those subjects who were prepared to participate representative of the entire population from which they were recruited?
7	Were the staff, places, and facilities where patients were treated representative of the treatment the majority of patients receive? Was PA in line with guidelines or a program ad-hoc? Internal validity
8	Were the main outcome measures used accurate (Is the device valuated and reliable)?
9	Was this study a clinical trial?
10	Was it randomized?
11	Was it blind?
12	Did the adherence have a direct output (e.g., it did not need to be derived)?
13	Was the output of the monitor in line with guidelines?
14	Was the duration of study in line with guidelines?
15	Was there a follow-up?

**Table 2:** Quality assessment and risk of bias

Study question	Backman et al. [51]	Benzo et al. [52]	Gary et al. [53]	Hoaas et al. [44]	Hoaas et al. [50]	Mendoza et al. [49]	Moy et al [54]	Pinto et al. [55]
1	1	1	1	1	1	1	1	1
2	1	0	1	0	1	1	1	1
3	1	1	1	1	1	1	1	1
4	0	0	1	0	1	1	1	1
5	0	0	1	0	1	1	1	1
6	0	0	0	0	0	0	0	0
7	0	0	0	0	0	0	0	0
8	1	1	0	0	1	1	1	1
9	0	1	0	1	1	1	1	0
10	1	0	1	0	0	1	0	1
11	0	0	0	0	0	1	0	0
12	1	1	1	1	1	0	0	1
13	1	1	1	1	1	1	1	1
14	0	0	1	1	1	1	0	1
15	0	0	1	1	1	0	0	0
Total	7	6	10	7	11	11	8	10
Score	Medium	Medium	Medium	Medium	High	High	Medium	Medium

## Results

**Fig. 1:**
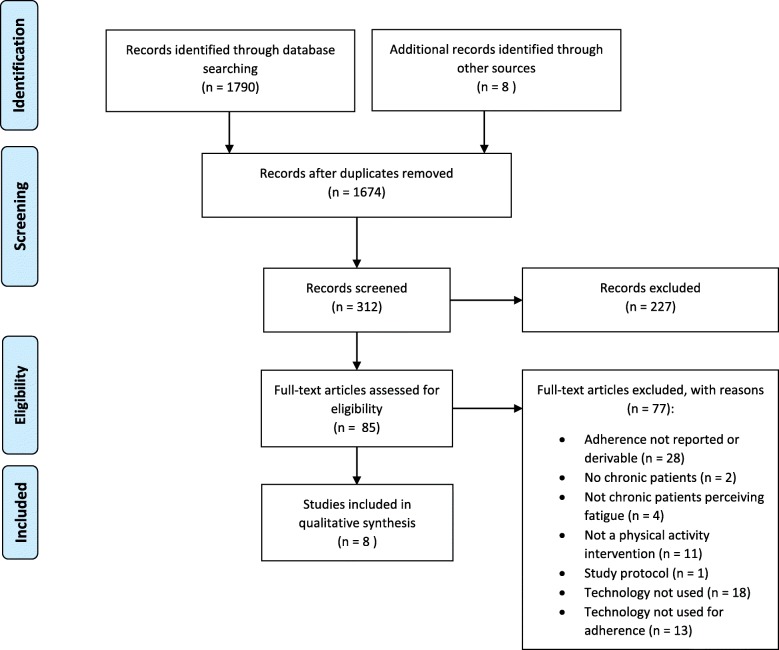
Selection process of studies

The study search process is presented in the Fig. 1. The search identified 1790 records, 1674 after removing duplicates. During the process of study selection and data analysis, eight more studies were identified through reference lists as possibly interesting and included. From records screened, we selected 312 abstracts. A total of 85 full-text manuscripts were reviewed. In the end, eight studies were selected based on our inclusion criteria and included in this systematic review. Two studies referred to the same data collection [44, 50]. Main outcomes of included studies are summarized in Table 3.

**Table 3:** Summary of the main data from the studies included

Study	Chronic disease	Number of participants	Control group	Timeline (weeks)	Mean age (years)	Sex M/F	Program’s goal	PA outcome	Adherence	Primary outcome of each study	Secondary outcomes of each study
Backman et al. [51]	Breast and colorectal cancer	39	Yes	10	54	8/31	10,000 steps	Step counts	≃ 90% week 1, ≃ 85% week 10^a^	Adherence to PA intervention	HRQoL, body composition and blood markers
Benzo et al. [52]	COPD	12	No	8	> 40	Not specified	12 min walk	Walking minutes	89 ± 0.22%	Program feasibility	WAI-SR
Gary et al. [53]	HF	24	Yes	12	60	12/12	Individualized: from 30 to 60 walking minutes, 3 times per week	Walking minutes, daily steps	83%	CS-PFP10	Muscle strength, HRQoL, functional capacity, disease severity levels
Hooas et al. [44, 50]	COPD	10	No	156	55	5/5	Individualized	Training sessions	69.1% year 1, 40.5% year 2, 13.9% year 3 (9/10)^b^	Adherence	System Usability Scale
Mendoza et al. [49]	COPD	50	Yes	13	68	33/17	Personalized	Step counts	80±31% week 4, 85 ± 26% week 8, 81 ± 22% week 13^c^	Daily step counts	Exercise capacity (6MWT), health status (SGRQ, CAT)
Moy et al. [54]	COPD	27	No	13	72	27/0	Personalized	Step counts	90.39% at baseline, 104.82% week 12^a^	Daily step counts	General health status, MMRC dyspnea score, Bristol knowledge, exercise self-efficacy
Pinto et al. [55]	Breast cancer survivors	43	Yes (but not analyzed)	12	53	0/43	Personalized	Exercise minutes, step counts	88.37% week 1, 55.81% week 12	Adherence	Exercise self-efficacy

*HRQoL* Health Related Quality of Life, *COPD* Chronic Obstructive Pulmonary Disease, *WAI-SR* Working Alliance Inventory‐Short Revised, *HF* Heart Failure, *CS-PFP10* Continuous Scale Physical Functional Performance test, *6MWT* 6-Min Walk Test, *SGRQ* Saint George’s Respiratory Questionnaire, CAT Chronic obstructive pulmonary disease Assessment Test, *MMRC* Modified Medical Research Council

^a^calculated by authors

^b^ at 3 year follow-up there were only 9 patients

^c^data requested and obtained from corresponding author

### Patient Groups

This systematic review includes eight studies, comprising 195 patients, 89 COPD [41, 45, 46, 48, 51], 39 cancer patients [51], 43 cancer survivors [55], and 24 patients with HF [53]. Mendoza et al. recruited COPD patients with both I, II, III, and IV GOLD 2011 [56] classification [49]. The same patients had no (*n* = 8), slight (*n* = 22), moderate (*n* = 14), and severe (*n* = 8) dyspnea and none had very severe dyspnea, assessed with the Modified Medical Research Council (MMRC) dyspnea scale [49]. Patients with other chronic conditions were excluded [49]. Moy et al. recruited patients with no (*n* = 4), slight (*n* = 10), moderate (*n* = 6), severe (*n* = 4), and very severe (*n* = 1) dyspnea assessed with the MMRC scale [54]. Benzo et al. recruited patients with II, III, and IV COPD GOLD 2011 [56] stage [52]. The description of the ten COPD patients undergoing 2 years intervention and one additional year of unsupervised follow-up [44, 50] is presented in Zanaboni et al. [57], showing that the majority of them had high COPD assessment test (CAT) scores (*n* = 6), and the rest medium (*n* = 3) or low (*n* = 1) CAT scores. Backman el al. recruited patients with breast and colorectal cancer (CRC), all of whom undergoing chemotherapy treatment [51]. Pinto et al. recruited 43 breast cancer survivors, with cancer at stage 0 (*n* = 8), 1 (*n* = 17), and 2 (*n* = 18); 55.8% of them received chemotherapy [55]. Gray et al. selected patients with stable systolic HF, with slight and marked limitation to PA, class II and III of the New York Association (NYHA) classification [53].

### Program Characteristics

In three studies, PA programs were based on step counts [49, 51, 54]; in other studies, the goal was expressed in minutes of activity [44, 50, 52, 53, 55]. PA programs were personalized and activity levels adjusted in four studies [49, 53–55], with the final goal to meet the guidelines formulated in the different studies. Hoaas et al. prescribed a home-based interval training based on heart rate, 4 × 4 min of intense walking, three times per week [44, 50]. Backman et al. proposed 10,000 steps per day as a fixed goal [51]. Benzo et al. used 12 min of slow walking in a COPD home-based rehabilitation program, 6 days/week [52]. In Backman et al., PA increased in the intervention group compared with the control group, but decreased during the study [51]. Benzo et al. did not analyze PA as pre-post intervention [52]. Programs lasted a minimum of 8 weeks to a maximum of 2 years.

### Monitoring Technology Characteristics

Pedometers were the prevalent type of monitoring technology used [49, 51, 54, 55]; other studies used activity monitors [52], pulse oximeters [44, 50], and heart monitors [53]. For seven out of eight studies, it was possible to determine the type of sensing technology used. The sensors were a 2-axial [53] and 3-axial accelerometer [49], a pair of piezoelectric sensors oriented at 90° [54], either a spring-levered or a piezo-electric pedometer [55], and finally an unspecified accelerometer [52]. The patients could check the last 7 days steps in all four pedometers, which kept around 30–40 days in memory. The activity monitor could store up to 21 days. Hoaas et al. [44] used a portable finger pulse oximeter (Nonin GO2 LED) to measure SPO_2_ and heart rate during exercise. This device does not have memory, thus patients were asked to write down their maximal HR, that they observed during every exercise session [57]. In Gary et al. in addition to the pedometer, a heart rate chest strap (Polar) was used [53]. Two devices had USB connection [51, 54], Vivofit 2 used in Benzo et al. had Bluetooth connectivity [52]; the DigiWalker, Omron HJ-112, the Tanita PD-724, and the Nonin GO2 LED did not seem to have any type of digital connectivity [49, 53, 55, 57]. Battery life ranged from 6 months to 1 year. All devices seemed to have a rather reasonable price, not exceeding £60 per unit (£24 to £58). Except for Hoaas et al. [44, 50] in which a £80 pulse oximeter, a £400 tablet, and a £600 treadmill were used per patient, making it up to a total of £1085 per patient in equipment cost only [57]. Two studies implemented PA monitoring on a website connected to the pedometers [52, 54]. PA monitoring was continuous for all studies.

### Monitoring Technology Usability

In Mendoza et al. and Backman et al., step counts measured by pedometers were manually reported in daily diaries [40, 52]. In contrast, in Benzo et al. and Moy et al., devices (i.e., pedometers and activity monitor armbands) were connected directly to an internet-supported digital system, so that patients could monitor their progress online [42, 53]. In Hoaas et al. [44, 50], videoconferences were used to remotely supervise training sessions, as the tablet was fixed onto the treadmill [57], during this conferences HR values were reported. In Gary et al. [53], the HF patients kept training session logs, and these were weekly reviewed by a nurse or an exercise specialist. Patients received monthly, or weekly, motivational support, in the form of calls, messages on a personal webpage, or e-mails in every study [44, 49–53, 55]. Motivational messages were shown to help patients increase their PA levels [58]. Benzo et al. and Backman et al. provided their patients with a fixed goal [51, 52]. Instead, in other three studies, the researchers gave periodical goals. Mendoza et al. set goals during weekly calls [52], Pinto et al. during monthly appointments [54], and Moy et al. delivered the first goal by e-mail; afterwards, patients could check their weekly goals directly from the website [53]. Hoaas et al.’s [44, 57] goals were set by using the rating of perceived exertion scale and the participants were motivated via videoconferencing [57].

Mendoza et al., Moy el al., and Pinto et al. provided personalized goals [52–54]. In these studies, patients’ PA increased. In fact, only Backman et al. did not report a PA increase; probably due to patients’ sufficiently active baseline levels (9000 steps/day) [40]. Only in Pinto et al. and Gary et al., the program was prescribed using heart rate monitoring to individualize moderate PA [53]. Goals in Gary et al. [53] were based on HR and RPE and progressively increased from 50 to 70% of their maximum.

Mendoza et al. considered pedometers user-friendly and reported experience of pedometer usability [52]. Conversely, Backman et al. observed that their patients experienced difficulties to manually record steps in activity diaries [40]. Moy et al. reported some problems in using pedometers and a website [53]. The main two problems were pedometer wearability at the waist, and first step counts upload; nonetheless, these difficulties were resolved in time.

### Fatigue

Of the selected studies, only Backman et al. quantified fatigue, assessed by means of the European Organization for Research and Treatment of Cancer quality of Life Questionnaire (EORTC QLQ-C30) [51]. Fatigue did not significantly increase during the intervention in this study and no differences were found compared to control group [51]. Although the other studies did not directly quantify fatigue, the patient populations investigated are known to suffer from fatigue, which exacerbates during exercise. Fatigue is in fact well characterized in all diseases included in this systematic review [59–62].

### Adherence Outcomes

Only two studies reported adherence as percentage of the total goal [51, 55]. Three reported it as the participation to PA session out of the total recommendation [44, 50, 53]. In the other studies, we derived adherence as follows. Mendoza et al. [49] provided us the data to calculate adherence. Then, we applied a one-way analysis of variance (ANOVA) for repeated measures to evaluate adherence values of the intervention group over time. Adherence values did not statistically differ. In the remaining studies [52, 54], adherence was calculated by us as a ratio between the executed PA and the PA goal (for details see adherence column Table 3).

## Discussion

The purpose of this systematic review was to evaluate the potential of using monitoring technology to improve adherence to a PA program, for patients with chronic diseases experiencing fatigue complaints. When looking at qualitative evidence provided by this systematic review, we concluded that it was not possible to establish whether the use of monitoring technology was able to positively influence physical activity adherence in patients with chronic diseases experiencing fatigue.

From 1790 hits, we included only eight studies. We selected the studies in which monitoring technology was used by patients to control their PA level and their progress and by doing so this would potentially improve program adherence. When defining the effect of monitoring technology to improve physical activity adherence, we included any technological tools which provided direct feedback to users (e.g., progress, achievements), as such able to increase their awareness and/or engagement, ultimately possibly resulting in a greater program adherence. We included only studies with patients with chronic diseases to whom fatigue could represent a serious obstacle to being active.

### Monitoring Technology Tools

The results underline that pedometers are the most used monitoring technology tools in the clinical context. The studies included in our systematic review seem to confirm that pedometers help to increase PA [63]. Unfortunately, an increase in PA was not always followed by health outcome improvements [40]. Although defining a PA program goal in steps is straightforward for most patients, this may in some cases not elicit a clear increase in their cardiorespiratory fitness. Moreover, half of the participants who reached a 10,000 steps target did not meet the minimum goal of 30 min of daily activity (in 10-min bouts) [64]. Programs that prescribed exercise intensity improved cardiorespiratory fitness more than programs prescribing only quantity of exercise (e.g., steps) [65]. Despite some difficulties, it could be inferred that the use of wearable devices, such as pedometers, is convenient and user-friendly for patients with chronic diseases even when they are elderly. However, it needs to be acknowledged that the accuracy of step counting at lower speeds has been found to be reduced [66, 67]. The only long-term adherence intervention study had, however, a small sample size (*n* = 10), and it did show a drastic decrease in adherence when the training sessions were no longer remotely supervised [44, 50]. This systematic review seems to point toward the idea that monitoring technology can help to reduce the gap between patients and therapists. Furthermore, it confirms the importance, and often the necessity, of maintaining a personal relationship through calls or personalized motivational messages. If these messages are impersonal and automated, this can lead to a lower efficacy [68].

### Drop-Outs and Fatigue

In Mendoza et al., drop-out rate was 4.9% (5 COPD patients, three in the intervention group and two in the control group). No specific causes were reported. Moy et al. described that three COPD patients did not complete the study (11%), reporting medical problems. Further, two patients were excluded from data analysis for incomplete step uploading, and interruptions due to medical problems [54]. Pinto et al. included the low rate of attrition (5%) as one of the strengths of their study [55]. In Backman et al., 26% of the cancer patients dropped-out, because of personal reasons, stress, treatments side effects, and fatigue [51]. For these patients, stress during treatment, health reasons, and fatigue constituted the barriers to nonparticipation. Despite the burdensome treatments, cancer patients showed a good or optimum PA level at the end of the interventions (≃ 8500 and 14,500 steps/day) [51, 55]. Indeed often, cancer patients did not have problems to complete PA interventions [69]. No dropouts were recorded by Gary et al. [53] during the intervention (*n* = 24) in HF patients. However, the screening of 615 potential participants led to a 75% rejection rate (documented non-adherence to medication was one of the exclusion criteria), and of the 25% finally invited only 4% responded to the invitation, enrolling a well-selected sample. Only one of the ten COPD patients in the study of Hoaas et al. [44, 50] dropped out during the 3-year study period.

An important selection criterion for this systematic review was the presence of fatigue suffered by the patients with chronic diseases undergoing a PA program. Although fatigue was known to be present in all eight studies selected, only Backman et al. assessed fatigue directly [51]. Fatigue was one of most relevant symptoms both for the intervention and the control group. Fatigue did not change during the study, yet according to Backman et al., it could be a possible cause of decrement in adherence [51].

PA levels of COPD patients were lower compared to cancer patients, yet the age of the COPD patients was higher compared to the other studies included in this systematic review and COPD conditions were moderate. This systematic review confirmed also that walking is the most common and feasible way to approach physical activity for patients with chronic diseases; in particular for cancer patients [69]. Only in Backman et al., colorectal cancer patients reported barriers to walking, as their therapy’s complications included hand-foot syndrome [51].

### Self-Efficacy and Intervention Duration

The importance of self-efficacy of patients with chronic diseases in carrying out a PA program is known [70]. In fact, Pinto et al. showed how self-efficacy values represent a predictor of goal achievement [55]. Interestingly, Moy et al. looked at self-efficacy at the beginning and at the end of the study and found no changes, whereas PA level increased [54]. Also in Hoaas et al. [44, 50], self-efficacy did not change from 2 to 3 years, while adherence dropped drastically. Backman et al. underlined how the length of the intervention influences adherence to a PA program; the longer the study, the lower the adherence [51]. Yet, Hoaas et al. outcomes seemed to suggest that cessation of remote supervision of training sessions was the underlying cause of the drop in adherence. The mean duration of the interventions considered in these studies was 32 weeks, ranging from 8 to 156 weeks*.*

### Clinical Outcomes

Backman et al. showed that cancer patients with a 10,000 steps goal for 10 weeks and adherence levels of 85%, decreased body weight without a change in body composition and decreased blood pressure, but did not show changes in inflammatory markers. Moreover, those cancer patients reported a decrease in symptoms but no alterations in QoL [51]. Moy et al. observed that COPD patients, undergoing 13 weeks personalized PA program with a very high adherence, did not change their general health status (measured with Medical Study Short Form-36 questionnaire) and neither their dyspnea [54]. Conversely, Mendoza et al. [49] with a similar program in similar patients as Moy et al. [54], but lower adherence, found changes in the health status (SGRQ and CAT) but not in dyspnea (MMRC). Yet in Mendoza et al. [49], absolute step counts were higher than in Moy et al. [54] (from 4000 to 7000 steps circa). Gary et al. [53] found a large increase in QoL (i.e., 23 point on the Minnesota Living With Heart Failure Questionnaire) in the intervention group, which had 83% adherence, versus no change in the control group. Hoaas et al. [44, 50] did not observe significant clinical changes in 2 years intervention but adherence after the first year was already below 50%. Benzo et al. and Pinto et al. did not report clinical outcomes and/or health status parameters [52, 55].

### Limitations

This systematic review has several limitations. One of the main limitations is the lack of adherence measurements the included literature analyzed. Moreover, there is poor consistency on what the term adherence really means when referring to PA programs. Pinto et al. and Backman et al. considered adherence both as continuous (minutes of exercise) and dichotomous (if participants met their goal) outcomes [51, 55]. Benzo et al., Gary et al., and Hoaas et al., reported adherence as a percentage of number of sessions performed, assuming that each daily session begun was completed [44, 50, 52]. The other two did not define adherence, which was calculated by us as the weekly mean of daily scores expressed as a percentage of the daily goal.

The low number of studies, the small sample size, the overall short duration of interventions (max 13 weeks), with the exception of one study, and the low heterogeneity of chronic disease types (only COPD, HF, and cancer patients) represent further limitations. At the same time, these limitations identify gaps in current literature regarding using wearable technology to stimulate an active lifestyle in special populations, based on which recommendations for future research directions can be determined. Finally, although this systematic review focused on patients with chronic diseases experiencing fatigue, fatigue was explicitly measured only in one study of the eight included. We have selected studies where, based on the literature, it was safe to assume the presence of fatigue complaints.

## Conclusions

The small body of evidence found in this systematic review does not allow us to establish whether the use of wearable technology was able to improve adherence to PA programs in patients with chronic diseases experiencing fatigue. The eight studies finally selected were of medium-high quality but with small sample sizes and including only three types of chronic diseases. In general, the studies analyzed in this systematic review showed high levels of adherence associated with monitoring technology, yet for rather short PA programs (max 13 weeks). Indeed the longest study taken into account, 2 years intervention plus 1 year follow-up showed a drop in adherence from 70% at the end of year 1 down to 40% at the end of year 2 [44]. Furthermore, in these studies, there did not seem to always be a strong relation between high adherence to PA programs and positive clinical health outcomes. This review has also underlined the necessity to clarify and standardize the definition of adherence. Six out of eight studies used very similar monitoring technology, predominantly pedometers, and all were worn at the waist. The adoption of mobile applications to monitor adherence in this population was still absent. Although these patients with chronic diseases experienced fatigue, this did not seem to influence adherence levels. Technology was mainly used to objectively monitor PA, rather than to improve adherence. Yet, the use of monitoring technology to improve fitness and wellbeing is a clear trend and may potentially be particularly useful as assistive tool to stimulate an active lifestyle and exercise adherence in special populations. Future research should investigate whether monitoring technology is effective in improving PA adherence, and if so, how effective it is and what the best implementation would be.

## Abbreviations

6MWT: 6-Minutes walking test
ACSM: American College of Sports Medicine
ANOVA: Analysis of variance
CAT: Chronic obstructive pulmonary disease assessment test
COPD: Chronic obstructive pulmonary disease
CS-PFP10: Continuous scale physical functional performance test
HF: Heart failure
HR: Heart rate
HRQoL: Health-related quality of life
MMRC: Modified Medical Research Council
PA: Physical activity
QoL: Quality of life
SGRQ: Saint George’s Respiratory Questionnaire
T2DM: Type 2 diabetes mellitus
WAI-SR: Working Alliance Inventory-Short Revised
